# Positional changes in the uvula tip after adenotonsillectomy in children: preliminary result

**DOI:** 10.7717/peerj.12243

**Published:** 2021-10-01

**Authors:** Suk Won Chang, Chan Il Song, Jeong Hong Kim, Gil-Chai Lim, Ju Wan Kang

**Affiliations:** 1 Department of Otorhinolaryngology, Jeju National University College of Medicine, Jeju, Republic of Korea; 2 Department of Otorhinolaryngology, Gangnam Severance Hospital, Yonsei University College of Medicine, Seoul, Republic of Korea

**Keywords:** Uvula, Children, Adenotonsillectomy, Soft palate

## Abstract

**Background:**

Adenotonsillectomy has become the primary treatment for children with sleep-disordered breathing or obstructive sleep apnea. However, few studies have investigated positional changes in the soft palate or uvula after adenotonsillectomy in children. The present study aimed to evaluate positional changes in the uvula tip using cephalometric analyses after adenotonsillectomy in children.

**Methods:**

We analyzed 160 pediatric patients from December 2015 to July 2016, with 94 pediatric patients who underwent adenotonsillectomy as the experimental group and 66 children who were treated and followed up with frequent tonsillitis as the control group. Positional changes in the uvula tip after surgery in the adenotonsillectomy group were investigated using lateral cephalograms obtained within 1 month before surgery and 3–4 months after surgery. Two lateral cephalogram intervals for a few months in the control group who did not undergo adenotonsillectomy were analyzed.

**Results:**

The palatal length (23.92 ± 3.47 *vs*. 24.46 ± 3.26; *p* = 0.032), palatal angle (43.13 ± 7.76 *vs*. 46.12 ± 5.91; *p* < 0.001), and retrouvula length (15.60 ± 3.51 *vs*. 16.60 ± 2.97; *p* = 0.009) were significantly increased on postoperative images relative to those on preoperative images. In the control group, there was a significant change in the palatal angle (2.99 ± 5.85 *vs*. 0.27 ± 4.14; *p* < 0.001) and retrouvula length (0.99 ± 3.64 *vs*. 0.05 ± 1.44; *p* = 0.025), but not in the palatal length (0.58 ± 2.38 *vs*. 0.043 ± 1.26; *p* = 0.065).

**Conclusion:**

The findings of the present study suggest that the uvula tip is displaced in the anteroinferior direction 3 or 4 months after adenotonsillectomy in children. Thus, clinicians should be aware that the retropalatal space may expand after adenotonsillectomy in the pediatric population.

## Introduction

Adenotonsillectomy is one of the most common pediatric surgeries performed in the United States, with more than 530,000 procedures performed annually in children aged <15 years (Ramos, Mukerji & Pine, 2013). Adenotonsillectomy was initially performed for recurrent tonsillitis; however, hyperplasia of the tonsils or adenoid may cause various problems in children, such as Eustachian tube dysfunction/otitis media, rhinosinusitis, obstructive sleep apnea (OSA), failure to thrive, dysphagia, halitosis, reduced ability to smell and taste, speech impairment, and abnormal dentofacial growth (Darrow & Siemens, 2002; Souki et al., 2010). Therefore, adenotonsillectomy has emerged as the primary treatment for children with sleep-disordered breathing or OSA (Mitchell, Pereira & Friedman, 2006).

Adenotonsillectomy can be performed using several techniques. The conventional technique for adenotonsillectomy involves “cold dissection” using metal instruments, with alternative surgical procedures including electrosurgery, laser dissection, dissection with a harmonic scalpel, argon plasma coagulation, and coblation. Each technique has its advantages and disadvantages, and the optimal technique remains unclear. The selection of the technique is generally based on the surgeon’s preference, training, and experience (Burton & Doree, 2017). However, preservation of anatomic structures, including the anterior and posterior pillars, after adenotonsillectomy, is considered important. A previous study showed that reduced anterior tonsillar pillar support after adenotonsillectomy predisposed patients to OSA syndrome (OSAS) (Chan, Akst & Eliachar, 2004).

Regarding wound healing after adenotonsillectomy, the tonsil fossae are covered with inflammatory exudates after tonsil removal, followed by the gradual replacement of the inflammatory debris and ingrowth of granulation tissue during the second week of recovery (Davidoss et al., 2018; Wexler, 1996). Additionally, the unification of the anterior and posterior pillars is often observed during the follow-up period.

Few studies have evaluated positional changes in the soft palate or uvula after adenotonsillectomy in children. Therefore, the present study aimed to investigate positional changes in the uvula tip (UT) using cephalometric analyses before and after adenotonsillectomy in children.

## Materials and Methods

### Participants

This study included pediatric patients aged 4–13 years who underwent adenotonsillectomy at between December 2015 and July 2016. Patients aged ≥ 13 years, those who did not undergo cephalometry, and those with dentofacial anomalies were excluded. Children, who were treated with frequent tonsillitis but refused adenotonsillectomy between December 2015 and July 2016, were enrolled as a control group. First lateral cephalogram, which was widely used to identify adenoid hyperptrophy, was obtained at first visit. However, the second examination was needed for the purpose of this study, therefore we are explained about this study to the parents or guardians of children. A second test was conducted on those who agreed to this study. We obtained the informed consent from the parents or guardians of all participation for the study. The post-operative or second lateral cephalogram were taken after the consent for this study. This study was approved by the Institutional Review Board of Jeju National University Hospital in accordance with the Declaration of Helsinki (JEJUNUH 2015-10-012).

### Cephalometric analyses

**Figure 1:**
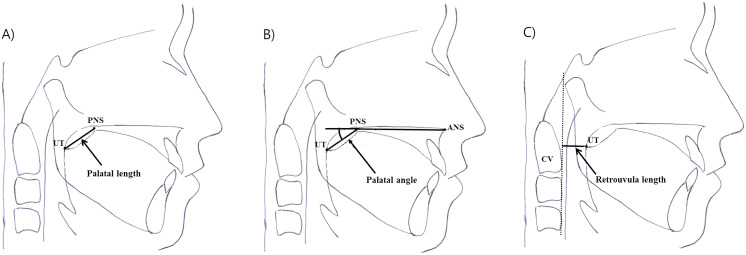
Measurement of three parameters using lateral cephalometric analyses before and after adenotonsillectomy in children. (A) Palatal length: the distance between the border of the posterior nasal spine (PNS) and the uvula tip (UT). (B) Palatal angle: the angle formed between the nasal floor and a line joining the PNS and UT. (C) Retrouvula length: the vertical distance between the UT and anterior border of the cervical spine.

Lateral cephalograms were obtained for all patients within 1 month before and 3–4 months after surgery in the adenotonsillectomy group. Two serial lateral cephalograms were recorded at an interval of a few months for the control group. All images were acquired using a standardized technique, in which the participant stood with the sagittal plane parallel to the film, with bilateral ear rods gently inserted into the external auditory meatus for stabilization of the head position during exposure. The jaws were maintained in centric relation, with the teeth in occlusion, lips relaxed, and head in the natural position (Chang & Chiang, 2017). We obtained accurate lateral cephalograms in children by an experienced radiological technologist. In children who did not cooperate reasonably, the radiological technologist and parents wore a radiation protective lead apron and helped the child position. Three parameters were measured, as described below (Fig. 1).

1) Palatal length: the distance between the border of the posterior nasal spine (PNS) and UT.
2) Palatal angle: the angle formed between the nasal floor and a line joining the PNS and UT.
3) Retrouvula length: the vertical distance between the UT and anterior border of the cervical spine.

Two researchers blinded to patient features and the study purpose measured each parameter and the mean value was used for analysis.

### Statistical analysis

Variables in pre-and postoperative measurement showed normal distribution in Kolmogorov–Smirnov test. Therefore, pre-and postoperative measurements were compared using paired t-tests. All statistical analyses were performed using SPSS (18.0.0; SPSS Inc., Chicago, IL, USA). A *p*-value of <0.05 (two-tailed) was considered statistically significant.

## Results

A total of 94 patients, including 54 boys and 40 girls aged 4–13 years (mean age, 7.2 ± 2.7 years), comprised the adenotonsillectomy group. The average time gap between the two cephalograms was 113 (standard deviation [SD] ± 21.1) days. A total of 66 patients who did not undergo adenotonsillectomy, comprising 38 boys and 28 girls with a mean age of 4.9 ± 2.0 years (2–9 years), were enrolled in the control group. The time gap between the two cephalograms was 99 (SD ± 31.8) days.

**Figure 2:**
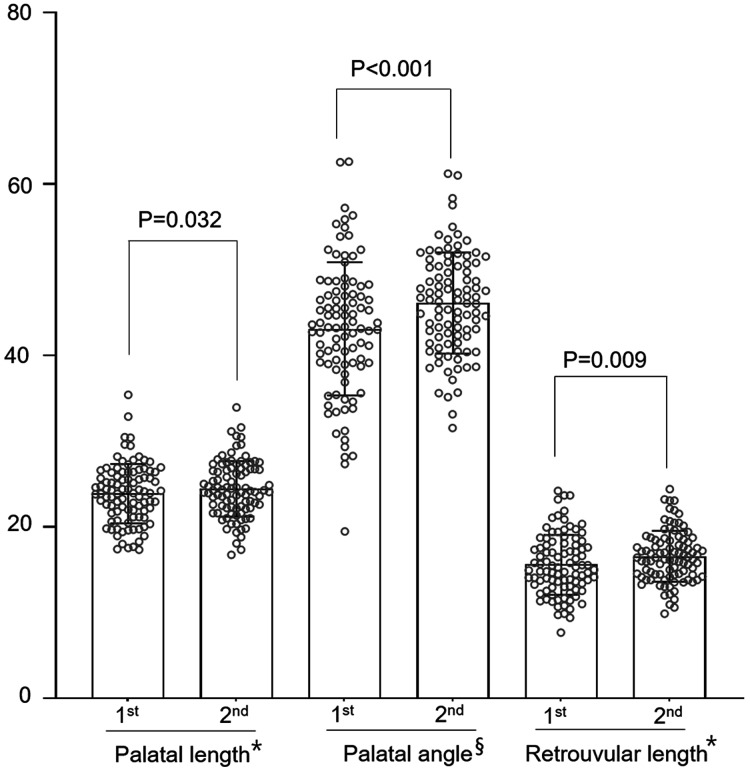
Comparison of parameters using lateral cephalograms obtained before and after adenotonsillectomy in children (*n* = 94). *For palatal length and retrouvular length, mm is used in unit. §For palatal angle, °is used in unit.

Figure 2 shows the results of the adenotonsillectomy group. Regarding palatal length, 53 and 41 patients showed an increase and decrease, respectively, after surgery. The overall mean palatal length was significantly greater after surgery (23.92 ± 3.47 mm) than before surgery (24.46 ± 3.26 mm; *p* = 0.032). The palatal angle increased in 67 patients and decreased in 27 patients after adenotonsillectomy. Overall, the palatal angle tended to increase after surgery, with a significant difference in the mean angles before (43.13° ± 7.76°) and after (46.12° ± 5.91°) surgery (*p* < 0.001). Finally, surgery increased and decreased the retrouvula length in 60 and 34 patients, respectively, with the mean postoperative value (16.60 ± 2.97 mm) being significantly greater than the preoperative value (15.60 ± 3.51 mm, *p* = 0.009).

**Figure 3:**
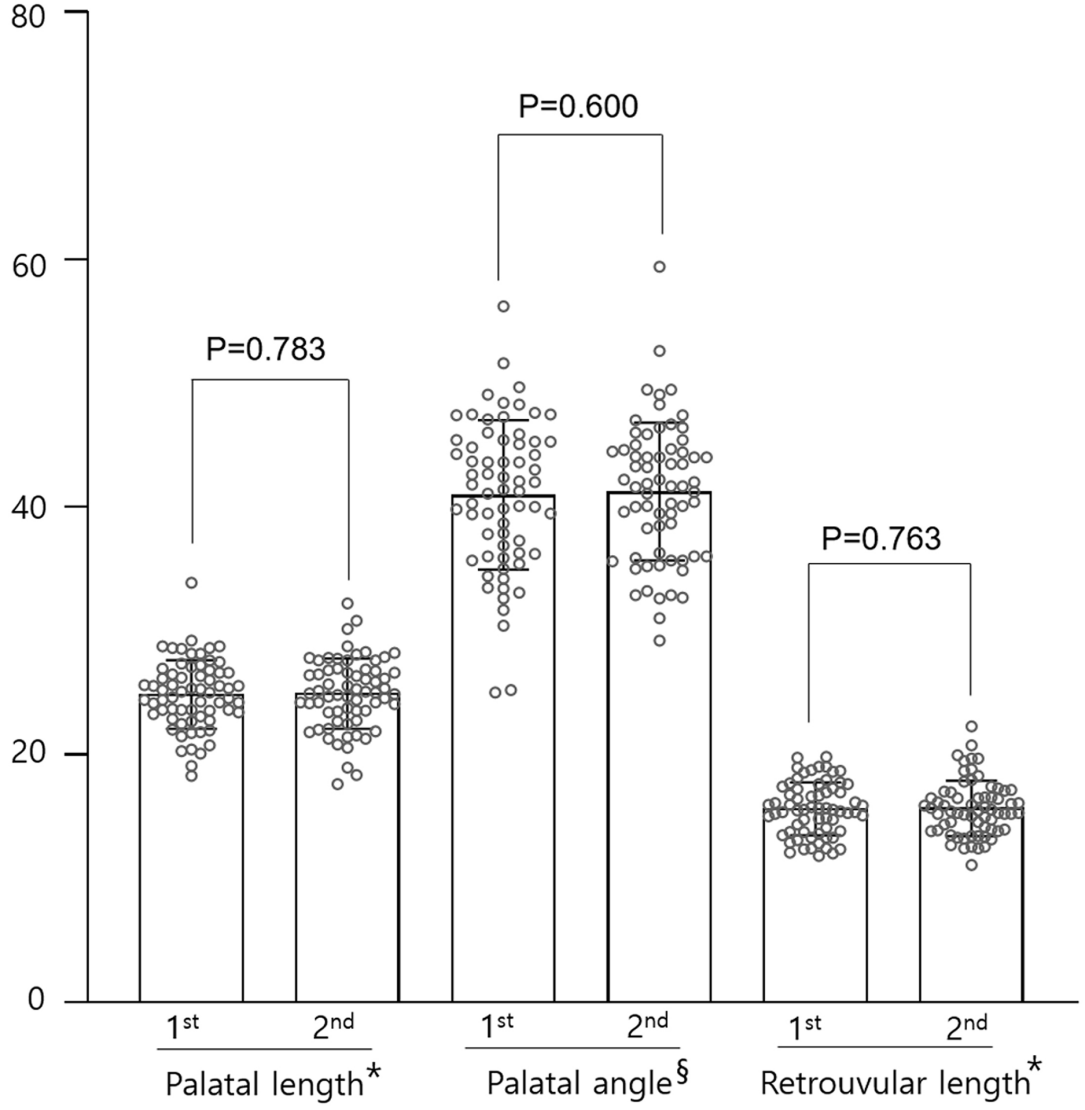
Comparison of parameters using cephalograms obtained at about 3-month intervals in the control group (*n* = 66). *For palatal length and retrouvular length, mm is used in unit. §For palatal angle, °is used in unit.

In the control group, palatal length increased in 37 patients and decreased in 29 patients. The average at the first measurement was 24.89 ± 2.76 mm and the second measurement was 24.93 ± 2.86 mm, and there was no significant change (*p* = 0.930, Fig. 3). The palatal angle increased and decreased in 35 and 31 patients, respectively. The mean first and second measurement values were (40.99° ± 6.02° and 41.26° ± 5.59°), respectively, and no significant difference was observed (*p* = 0.791, Fig. 3). Retrouvula length increased in 38 patients and decreased in 29 patients. The average at the first measurement was 15.64 ± 2.14 mm, and the second measurement was 15.69 ± 2.23 mm. There was no significant change (*p* = 0.888, Fig. 3).

A comparison of the changes between the two groups showed that the mean of the palatal length change was (0.58 ± 2.38 mm) in the adenotonsillectomy group and (0.043 ± 1.26 mm) in the control group. Moreover, there were no significant differences (*p* = 0.065, Table 1). The average palatal angle change was significantly increased in the adenotonsillectomy group (2.99° ± 5.85°) compared to that in the control group (0.27° ± 4.14°, *p* < 0.001, Table 1). In addition, a significant change was observed in the retrouvula length between the adenotonsillectomy and the control groups (*p* = 0.025, Table 1). The mean of the retrouvula length change was (0.99 ± 3.64 mm) in the adenotonsillectomy group and (0.05 ± 1.4 mm) in the control group.

**Table 1:** Comparison of changes of parameters in two groups.

	Adenotonsillectomy group (*n* = 94)	Control group (*n* = 66)	*p*-value
Palatal length change	0.58 ± 2.38	0.043 ± 1.26	0.065
Palatal angle change	2.99 ± 5.85	0.27 ± 4.14	<0.001
Retrouvula length change	0.99 ± 3.64	0.05 ± 1.44	0.025

**Notes:**

Palatal length change: change in two serial cephalograms of the palatal length.

Palatal angle change: change in two serial cephalograms of the palatal angle.

Retrouvula length change: change in two serial cephalograms of the retrouvula length.

## Discussion

In the present study, we used cephalometric analyses to demonstrate that the UT is displaced in the anteroinferior direction 3 or 4 months after adenotonsillectomy in children.

Cephalometric radiographs were used to evaluate facial growth and development. Cephalometric analysis has become a popular and inexpensive tool for the detection of obstruction sites in patients with OSAS (Chang & Chiang, 2017). Cephalometry can be used for soft tissue evaluation at low radiation doses, and the findings correlate with those of other modes of investigation, such as computed tomography (CT). In particular, the posterior airway space measured using lateral cephalometry was found to be strongly correlated with that measured using three-dimensional CT, with high accuracy and predictability (Samman, Mohammadi & Xia, 2003).

Adenotonsillectomy was traditionally performed for recurrent tonsillitis. Nowadays, it is also used for the treatment of OSAS in children as studies have shown that children with OSAS tend to exhibit vertical facial growth, crossbite, increased overjet, and narrowing of dentition. Additionally, impaired endothelial function and cardiovascular autonomic activity have been observed in pediatric patients with OSAS (Chang & Chiang, 2017). Adenotonsillectomy, when performed in a timely manner, can prevent these problems.

Fluoroscopic studies in children have shown that obstruction is caused by the forward movement of the posterior pharyngeal wall, downward and posterior movement of the tonsils, and/or medial movement of the lateral pharyngeal walls. In a previous study, adenotonsillectomy significantly ameliorated OSA symptoms in children with anatomical changes in the upper airway (Suen, Arnold & Brooks, 1995). Although multiple factors have been implicated in the pathogenesis of OSAS in children, a better response to adenotonsillectomy suggests that anatomic factors predominate in the pediatric population.

In the present study, we found that the palatal length, palatal angle, and retrouvula length were significantly increased after adenotonsillectomy in children, with the findings indicating an anteroinferior positional change at the UT 3 or 4 months after the surgery. And, the size of tonsils and adenoids is thought to have no significant effect on the change in these variables after adenotonsillectomy (Supplemental_2). However, there were no significant changes between the two serial tests in the control group. When we compared the change in variables tested at approximately 3-month intervals in the two groups, significant changes in the palatal angle and the retrouvula length were observed in the adenotonsillectomy group. In addition, the palatal length change was increased in the adenotonsillectomy group, although the difference was not significant.

Therefore, we postulated that adenotonsillectomy affected the position of the UT. Moreover, the unification of the anterior and posterior pillars is commonly observed during the follow-up period after adenotonsillectomy. We speculate that this adhesion influences the movement of the uvula, with subsequent positional changes leading to the expansion of the retropalatal space. Our results of expanded retropalatal space support the results of previous studies that adeno-tonsillectomy might be suggested as the first treatment method in children with obstructive sleep apnea.

To the best of our knowledge, the present study is the first to evaluate positional changes in the uvula after adenotonsillectomy in children. Previously, Chan, Akst & Eliachar (2004) reported the posterior collapse of the soft palate after adenotonsillectomy and suggested that this phenomenon occurred because of the loss of anterior pillar support. Thus, the authors emphasized the importance of preserving the anterior pillar (Chan, Akst & Eliachar, 2004). We preserved the anterior pillar in all the patients. As aforementioned, this preserved pillar generally unifies with the posterior pillar and leads to positional changes in the uvula.

This study has some limitations. First, cephalograms are two-dimensional images that do not allow volumetric assessments. Nevertheless, they demonstrated anteroinferior positional changes in the UT. Second, the follow-up period was short, and long-term outcomes were not evaluated. Third, the ages of participants in the adenotonsillectomy and control groups and the interval between the two cephalograms were not entirely consistent, making accurate comparison difficult. The disease entities of the two groups were also different, and we should consider children with hypertrophy of the tonsils and adenoid as the control group for an exact comparison between the two groups. More careful consideration about demographic factors like age, sex, and disease entity will be needed in future studies. Four, we have showed the possibility of changing the location of the uvula after adenotonsillectomy, however there is still a lack of content about clinical significance. Consequently, further studies of clinical significance are thought to be necessary in future studies. Finally, the sample size was inadequate to lead to a strong conclusion.

The findings of the present study suggest that the UT is displaced in the anteroinferior direction 3 or 4 months after adenotonsillectomy in children. Thus, clinicians should be aware that the retropalatal space may expand after adenotonsillectomy in the pediatric population. Further investigations are necessary to understand these positional changes and soft palate conditions, as well as the clinical significance of these changes after adenotonsillectomy in children.

## Supplemental Information

10.7717/peerj.12243/supp-1 Supplemental Information 1 Raw data. Three parameters (palatal length, palatal angle, and retrouvula length) using lateral cephalometric analyses, sex, and age in children with or without adenotonsillectomy.

10.7717/peerj.12243/supp-2 Supplemental Information 2 Comparison of parameters using lateral cephalograms obtained before and after adenotonsillectomy in children according to grade of tonsil size and adenoid size. *Unfortunately, in one out of 94 children, we could not determine the size of the tonsils and adenoids, so we analyzed 93 children except for one.

## Additional Information and Declarations

### Data Availability

The following information was supplied regarding data availability: The raw measurements are available in the Supplementary Files.
