# Supplementary material for: Positional changes in the uvula tip after adenotonsillectomy in children: preliminary result
Source: PeerJ. 2021 Oct 1;9:e12243. doi: 10.7717/peerj.12243 (PMC8489408; doi:10.7717/peerj.12243)
Supplement: Supplemental Information 2 — *Unfortunately, in one out of 94 children, we could not determine the size of the tonsils and adenoids, so we analyzed 93 children except for one. [file peerj-09-12243-s002.docx]

Supplemental table 1. Comparison of parameters using lateral cephalograms obtained before and after adenotonsillectomy in children according to grade of tonsil size.

|  | Tonsil Grade | | | p-value |
| --- | --- | --- | --- | --- |
|  | Gr 2 (n=10) | Gr 3(n=61) | Gr 4 (n=22) |  |
| Palatal length | 0.4 ± 2.4 | 0.4 ± 2.5 | 1.1 ± 2.1 | 0.564 |
| Palatal angle | 3.1 ± 4.4 | 3.1 ± 5.7 | 3.0 ± 7.0 | 1.000 |
| Retrouvular length | 0.9 ± 2.4 | 1.0 ± 3.6 | 1.0 ± 4.4 | 0.999 |

*Unfortunately, in 1 out of 94 children, we could not determine the size of the tonsils and adenoids, so we analyzed 93 children except for one.

**Grade 1 – Tonsils are entirely within the tonsillar pillar. Grade 2 – Tonsils are extending to the pillars less than 50% of the lateral dimension of the oropharynx. Grade 3 – Tonsils are extending to the between 50% and 75% of the lateral dimension of the oropharynx. Grade 4 – Tonsils occupy more than 75% of the lateral dimension of the oropharynx.

Supplemental table 2. Comparison of parameters using lateral cephalograms obtained before and after adenotonsillectomy in children according to grade of adenoid size.

|  | Adenoid Grade | | | | p-value |
| --- | --- | --- | --- | --- | --- |
|  | Gr 1 (n=2) | Gr 2 (n=37) | Gr 3 (n=46) | Gr 4 (n=8) |  |
| Palatal length | 2.6 ± 6.9 | 0.8 ± 2.1 | 0.6 ± 2.4 | -0.9 ± 1.7 | 0.190 |
| Palatal angle | 2.3 ± 1.7 | 4.0 ± 5.0 | 2.4 ± 6.7 | 2.8 ± 4.5 | 0.639 |
| Retrouvular length | 6.8 ± 1.4 | 1.3 ± 2.7 | 0.5 ± 4.2 | 0.6 ± 3.2 | 0.101 |

*Unfortunately, in 1 out of 94 children, we could not determine the size of the tonsils and adenoids, so we analyzed 93 children except for one.

**Grade 1 – Adenoids tissue filling one-third of the vertical portion of the choanae. Grade 2 – Adenoids tissue filling from one-third to two-third of the choanae. Grade 3 – From two-third to nearly complete obstruction of the choanae. Grade 4 – Complete choanal obstruction.
